# Microbial Character Related Sulfur Cycle under Dynamic Environmental Factors Based on the Microbial Population Analysis in Sewerage System

**DOI:** 10.3389/fmicb.2017.00064

**Published:** 2017-02-14

**Authors:** Qian Dong, Hanchang Shi, Yanchen Liu

**Affiliations:** State Key Joint Laboratory of Environment Simulation and Pollution Control, State Environmental Protection Key Laboratory of Microorganism Application and Risk Control, School of Environment, Tsinghua UniversityBeijing, China

**Keywords:** sewerage system, microbial structure, sulfur cycle, sulfate-reducing bacteria, sulfur-oxidizing bacteria

## Abstract

The undesired sulfur cycle derived by microbial population can ultimately causes the serious problems of sewerage systems. However, the microbial community characters under dynamic environment factors in actual sewerage system is still not enough. This current study aimed to character the distributions and compositions of microbial communities that participate in the sulfur cycle under the dynamic environmental conditions in a local sewerage system. To accomplish this, microbial community compositions were assessed using 454 high-throughput sequencing (16S rDNA) combined with dsrB gene-based denaturing gradient gel electrophoresis. The results indicated that a higher diversity of microbial species was present at locations in sewers with high concentrations of H_2_S. *Actinobacteria* and *Proteobacteria* were dominant in the sewerage system, while *Actinobacteria* alone were dominant in regions with high concentrations of H_2_S. Specifically, the unique operational taxonomic units could aid to characterize the distinct microbial communities within a sewerage manhole. The proportion of sulfate-reducing bacteria, each sulfur-oxidizing bacteria (SOB) were strongly correlated with the liquid parameters (DO, ORP, COD, Sulfide, NH_3_-N), while the *Mycobacterium* and *Acidophilic SOB* (M&A) was strongly correlated with gaseous factors within the sewer, such as H_2_S, CH_4_, and CO. Identifying the distributions and proportions of critical microbial communities within sewerage systems could provide insights into how the microbial sulfur cycle is affected by the dynamic environmental conditions that exist in sewers and might be useful for explaining the potential sewerage problems.

## Introduction

Sewerage system undertakes a significant role in the collection and transportation of wastewater in urban city. Complex microbial biochemical processes occur in sewerage pipes as the specific environment and abundant nutrient in wastewater. These microbes produce some negative effects, particularly undesirable biochemical sulfur processes that can cause serious problems of sewerage system. For instance, biogenic sulfuric acid can react with concrete, causing serious corrosion that could eventually result in the structural failure of the sewerage system (Zhang et al., 2008). In some cases, the degradation rate of the concrete can reach up to five millimeters per year around the sewerage level of the sewer pipes (Mori et al., 1992; Belie et al., 2004; Ling et al., 2014). Huge financial and energy costs need to be invested to maintain and/or replace damaged sewerage pipes. A recent study calculated the cost of wastewater system maintenance as approximately 4.5 billion dollars annually in USA, while the cost to replace 8000 miles of sewerage pipes is approximately 3.3 billion dollars (Carpenter, 2009). As microbial populations play a vital role in processing sulfur in sewerage systems, the characteristics of functional bacteria will determine the dynamic biochemical processes presented in the system. Therefore, gaining an understanding of the distinct bacterial populations related sulfur process in sewerage systems can aid odor control and help mitigate problems with sewerage system corrosion.

Hydrogen sulfide, which is produced by sulfate-reducing bacteria (SRB) under anaerobic conditions in sediments or biofilms at the bottom of sewerage pipeline, diffuse into a thin liquid films at the crowns of the pipelines in sewerage systems. Hydrogen sulfide and other sulfur compounds, such as S_2_O_3_^-^ and S^0^, are then oxidized biotically or abiotically, such that sulfate-producing bacteria accumulate on the pipeline surface (Okabe et al., 2007; Satoh et al., 2009; Soleimani et al., 2013; Ling et al., 2014). Calcium, silicon, and aluminum oxides, and carbonates in cured cement react with biogenic sulfuric acid, causing the corrosion of gypsum into ettringite, and resulting in a continuous decline in pH value on the surface (Vincke et al., 2001). Concrete surface is typically not a suitable location for the accumulation of most bacteria because of its pH changes substantially during the corrosion process. Therefore, a succession of different species of sulfur cycle bacteria was reported growing on the concrete surface in the form of biofilms. SRB initiates the sulfur cycle in a certain sense and was a primary H_2_S producer. Researchers have reported several solutions to prevent the formation of the anaerobic conditions that support the growth of SRB; these include the injection of O_2_ and the addition of other oxidants (e.g., nitrate and nitrite) (Gutierrez et al., 2008). Sufur-oxidizing bacteria (SOB), which play a key role in causing microbiologically influenced concrete corrosion (MICC), can be divided into two main groups: neutrophilic sulfur-oxidizing bacteria (NSOB) and acidophilic sulfur-oxidizing bacteria (ASOB) (Islander et al., 1991). *Thiothrix* sp., *Thiobacillus plumbophilus, Thiomonas intermedia, Halothiobacillus neapolitanus, Acidiphilium acidophilum* and *Acidithiobacillus*, and *A. thiooxidans* accounted for approximately 70% of EUB338-mixed probe-hybridized cells and was the most dominant SOB in the heavily corroded concrete sample (Okabe et al., 2007). ASOB, such as *Thiobacillus thiooxidans* and *Thiobacillus intermedius*, are primarily responsible for concrete structural failure of concrete structures in sewerage systems because they can survive in low pH conditions and have the ability to generate a highly acidic environment (Mori et al., 1992; Soleimani et al., 2013). In addition, *Mycobacterium* spp. and other acidophilic populations, such as some acidophilic SOB (M&A) have been found on a seriously corroded concrete surfaces under low pH conditions and are present during the final stages of corrosion (Vincke et al., 2001). The Biswas’s study showed that the biofilm was successively developed during winter and summer period (Biswas et al., 2014), which meant that SRB and SOB microbial structure would not change greatly over a short period of time.

However, there always present dynamic environment factors in the sewerage system. The microbial transformation of inorganics and organic molecules during the anaerobic-anoxic-aerobic wastewater processes occurs during wastewater conveyance. This process alters bacterial metabolism and produces different functional biomasses with varying characteristics (Hvitved et al., 1995; Raunkjar et al., 1995). Corrosion and odor problems mainly resulted from the generation of hydrogen sulfide and the subsequent microbial activity needed to process the sulfide into sulfuric acid (Hao et al., 1996). The rapid velocity of the fluid flow in sewerage systems prevents the deposition of sediments, creating a non-ideal habitat for SRB. Additionally, exposure to dissolved oxygen and nitrate exposure prevents the formation and emission of sulfide in sewers under anaerobic conditions (Nielsen et al., 2008). Surface neutralization and H_2_S oxidation at the early stages of sewer corrosion are influenced by H_2_S concentration, temperature and relative humidity (Jaseph et al., 2012). The microbial community structure can be greatly affected by environmental conditions. Moreover, the complex and dynamic process facilitates alternative aerobic and anaerobic conditions within drainage pipes based on spatial dimensions and temporal factors. The complicated microbial biochemical processes associated with the sulfur cycle vary with the liquid conditions in sewer operations and significantly affect the distribution of microbial communities in the sewerage system. However, the dominant functional microbial communities that participate in the sensitive biochemical reactions associated with the sulfur cycle would be whether somewhat more stable under the dynamic aerobic and anaerobic conditions coupled with water level fluctuations in sewers. The current understanding of how the dynamic fluctuations affect the distributions of dominant functional microbial communities associated with sulfur cycle remains limited and needs to be improved. As the microbial community accumulated in a sewerage pipe or manhole would be more stable under the dynamically changing environmental conditions, the distinct shifts in the microbial structure could rather implicate the potential prospective sulfur cycle in response to dynamic environmental factors in sewerage systems.

Relevant researches on functional microbial community in the actual sewerage system were rarely studied based on the technology of Next Generation Sequencing (Satoh et al., 2009; Hooper et al., 2010). Thus, the knowledge of the distribution of uncultured functional microbial communities based on the pyro-sequencing associated with the sulfur cycle and the correlation with environmental factors in sewer pipes could supplement the vacancy of this research area. Therefore, the study aimed to characterize the structures of the functional microbial communities following the prospective sulfur cycle in response to the dynamic environmental parameters in the sewerage system. The study was conducted in a local residential field of sewerage system. The samples collected in different spatial scale were examined at high resolution to determine community structures using next-generation sequencing technology, and the environmental parameters in sewerage pipe also were detected simultaneously. The distribution and shift of core microbial community signature within the sewerage system respond to the typical dynamic environmental factors were estimated. We attempt to link core microbial population and functional population to conventional sewerage environmental factors, such as H_2_S, sulfide, and COD, etc.

## Materials and Methods

### Sampling and Total DNA Extraction from Sediments

**FIGURE 1:**
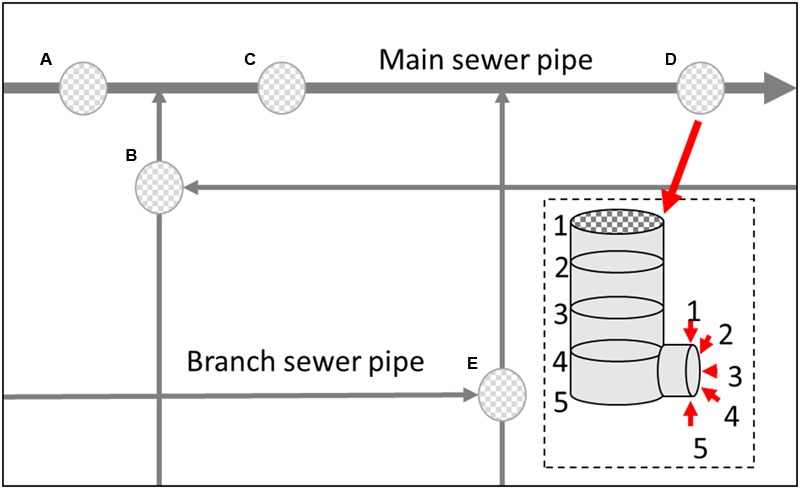
**Schematic showing the sampling site location within the experimental area (Wuxi, China)**.

**FIGURE 2:**
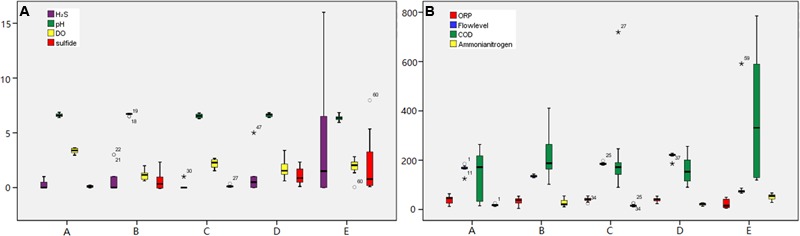
**Dynamic monitoring for the environmental parameters at each sampling site over 24-h.** The monitoring environmental factors were as follows: **(A)** H_2_S-ppm; pH; DO-mg/L; Sulfide-mg/L, **(B)** ORP-mV; Flowlevel-cm; COD-mg/L; Ammonia nitrogen-mg/L.

Samples were collected from different vertical location (numbered 1–5) within a manhole walls and connected sewerage pipelines (**Figure 1**) in both the main sewerage line and a branch sewerage line in a residential area of Wuxi, South China. The 13-day average values of fluctuation of environmental parameters in the gas and liquid phase in the sewerage system are shown in **Figure 2**. Microbial samples were collected with a scoop by scraping the sediments adhere on the manhole wall and the sewerage pipe as shown in **Figure 1**. As the dynamic changes of water level, samples were collected on submerged surfaces or unsubmerged surfaces. Then they were transferred into sterile centrifuge tubes by plumbers in the manhole, and transported to the laboratory and stored at -20°C. To enrich the microorganisms, those samples were centrifuged at 12000 rpm for 5 min and the supernatants were eliminated. Total DNA was extracted using a Fast DNA Spin Kit for Soil (MP Biomedicals, LLC) following the manufacturer’s instructions. DNA was extracted from three duplicate samples at each sampling site and then they were all merged together for the further study. Then, the extracted DNA solution was purified via ethanol precipitation, which has been proven to be especially effective for the samples from sewers in this study. The purified DNA solutions were then stored at -20°C for further analysis.

### PCR and Denaturing Gradient Gel Electrophoresis (DGGE) Analysis of SRB Using the dsrB Gene

A total of 50 samples were prepared to analyze the dsrB gene using the denaturing gradient gel electrophoresis (DGGE) method. Polymerase chain reaction (PCR) was performed in a 50 μl reaction, containing 0.5 μl of each primer (10 μM each), 5 μl of 10 X PCR buffer (MgCl_2_-free), 5 μl of MgCl_2_ (2.5 mmol/L), 0.2 μl of Taq DNA polymerase (5 U, TaKaRa Mirus Bio Corp., Madison, WI, USA), 4 μl of dNTP mix (2.5 mmol/L), 1 μl of dimethyl sulfoxide (DMSO), 31.8 μl of ultra-pure water and 2 μl of DNA template in each reaction. A 390-bp fragment targeting β-subunit of the dissimilatory sulfite reductase gene (dsrB) was amplified with the following primer pairs: primer 2060-GC (5′-CAACATCGTYCAYACCCAGGG-3′ with a GC-rich tail CGCCCGCCGCGCGCGGCGGGCGGGGCGGGGGC) and primer DSR4R (5′-GTGTAGCAGTTACCGCA-3′) (Mizuno et al., 2012). Apr (adenosine-5′-phosphosulfate reductase) gene was applied with the primers aprA-1-F-GC (5′-TGGCAGATCATGATYMATGG-3′) and aprA-5-R (5′-GCCCCAACYGGRCCRTA-3′) (Meyer and Kuever, 2007). A touch-down protocol was used with this primer pair as follows: 5 min at 95°C, 20 cycles of 40 s at 95°C, 30 s at each cycle during the touch-down annealing process of 50–60°C, and 1 min at 72°C; and then 29 cycles of 40 s at 95°C, 30 s at 50°C, and 1 min at 72°C; and finally, 10 min at 72°C for a final extension. The DGGE was performed using the DCode ^TM^ System (Bio-Rad Laboratories, Hercules, CA, USA). The PCR products were run on 8% denaturing gradient polyacrylamide gels with 30–60% of urea-formamide solution for 6.5 h. The analysis of DGGE fingerprints was conducted by using the Quantity One software. Microbial diversity was evaluated by calculating the Shannon-Wiener index of each sample (Dong and Reddy, 2010).

### 454 Pyro-sequencing of 16S rDNA

Next-generation DNA sequencing can provide in-depth descriptions of microbial communities. Samples were prepared to analyze the microbial community structures based on next-generation sequencing of the V3 region in samples collected from upper site 1 and lower site 4 of the sampling locations A and E, respectively. The following pair of primers was used to amplify the hypervariable V3 region (approximately 500 bp): 27F-5′-AGAGTTTGATCCTGGCTCAG-3′ and 533r-5′-TTACCGCGGCTGCTGGCAC-3′. The samples were individually barcoded to enable multiplex sequencing. PCR reactions were conducted in a 20-μl volume: 4 μl of 5× FastPfu Buffer; 2 μl of dNTPs (2.5 mM); 0.4 μl of forward primer (5 μM), 0.4 μl of reverse primer (5 μM), 0.4 μl of Fastpfu polymerase and 10 ng of template DNA. The following amplification protocol was used: 5 min initial denaturation at 94°C; 11 cycles of denaturation at 95°C, annealing at 60°C for 30 s, and extension at 72°C for 1 min; 25 cycles of denaturation at 95°C, annealing at 50°C for 30 s, and extension at 72°C for 1 min, and finally extension at 72°C for 10 min. DNA molecules marked with different barcodes were sequenced using a Roche Genome Sequencer FLX+ (Roche 454 Life Sciences, Branford, CT, USA) at Shanghai Majorbio Bio-pharm Technology, Co., Ltd.

### Sequence Analysis and Submission

The obtained sequencing data were denoised, and low quality sequences, pyrosequencing errors and chimeras were removed using Qiime (Caporaso et al., 2010) (version 1.17)^1^. The remaining sequences were clustered into operational taxonomic units (OTUs) at 97% identity using Usearch (version 7.1)^2^. Simpson and Shannon-Wiener diversity indices were calculated using normalized data to reduce over-inflation in the pyrosequencing datasets (Gihring et al., 2012). The sequences generated in this study have been deposited into the short-reads archive database (Accession ID: SRX764278).

### Statistical Analysis Based on the 454 Pyro-sequencing Results

Redundancy analysis (RDA) and canonical correspondence analysis (CCA) were performed as previously described (Cayford et al., 2012). Strength correlation and the significance of the relationships that existed between different microbial communities, including those formed by SRB, SOB, and M&A groups, and the dynamic environmental factors were evaluated by using RDA/CCA and Spearman analysis.

## Results

### Evaluation of Microbial Diversity Exhibited by dsrB Gene-DGGE Fingerprints and 16S rDNA-Pyrosequencing

**FIGURE 3:**
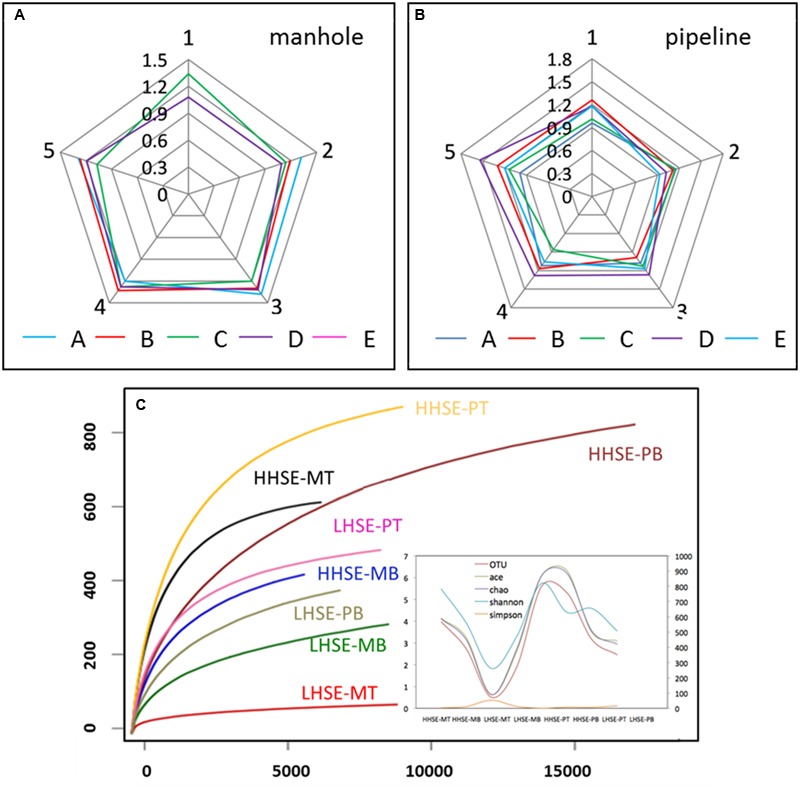
**Microbial diversity obtained by SRB-DGGE (A,B)** and 16S rDNA pyrosequencing **(C)** (MA, MB, MC, MD and ME represent the manhole samples; PA, PB, PC, PD, and PE represent the pipeline samples; and 1, 2, 3, 4, and 5 represent the spatial locations; PT: top of the pipeline; PB: bottom of the pipeline; MT: top of the manhole; MB: bottom of the manhole).

The samples were collected from the five vertical sites of each manhole and pipeline were pre-examined using DGGE. The results showed that the SRB microbial diversity in each sample mainly distributed between 1.0 and 1.6, which was a relatively concentrated in microbial-diversity fingerprints (**Figures 3A,B**; **Supplementary Figure S1**). The variation of the SRB diversity from each location was not significant; however, this variability was clearly different from the regularities of the whole microbial diversity detected by 16S rDNA sequencing in our previous study (Liu et al., 2015). The similarities in the distribution of the SRB diversity might be caused by SRB possess much stronger resistance to sewer environmental conditions than the other bacteria groups. The detected SRB population of each sample mainly included *Desulfobacter* sp. and *Desulfobulbus* sp. The DGGE results also indicated that SRB diversity was similar across the top and bottom sides of the pipe; however, there was much higher diversity in the bottom portion relative to the top portion of the manhole. In contrast, SOB could not be amplified by the primer pair used, suggesting that they existed at quantities that were hard to be detected. To further assess the community compositions of the SRB and SOB in pipeline, samples were collected from two locations for pyrosequencing. Location E was selected as a representative high hydrogen sulfide exposure (HHSE) site, while location A was selected as a representative low hydrogen sulfide exposure (LHSE) site. Based on the 16S rDNA libraries that were constructed using the results from the 454 pyrosequencing, the rarefaction curves of the eight samples at distance cutoff levels of 3% demonstrated that the bacterial phylotype richness was higher in the HHSE location than that in the LHSE location, regardless of whether the samples were collected from the sewerage pipe or the manhole (**Figure 3C**; **Supplementary Figure S2**). The Shannon diversity index was used to estimate the microbial community diversity in the samples based on the abundance and evenness of the population distribution. The Shannon index for the HHSE sample was larger than that for the LHSE sample, suggesting that more abundant and consistent OTUs exist in the environments with a high H_2_S concentration relative to those with a low H_2_S concentration environment. Moreover, there was much higher microbial diversity in the sewerage pipe relative to the manhole and a higher diverse microbial community exists when high concentrations of H_2_S are present in the sewerage system (**Figure 3C**).

### Taxonomic Distribution of OTUs and Microbial Community Structure

**FIGURE 4:**
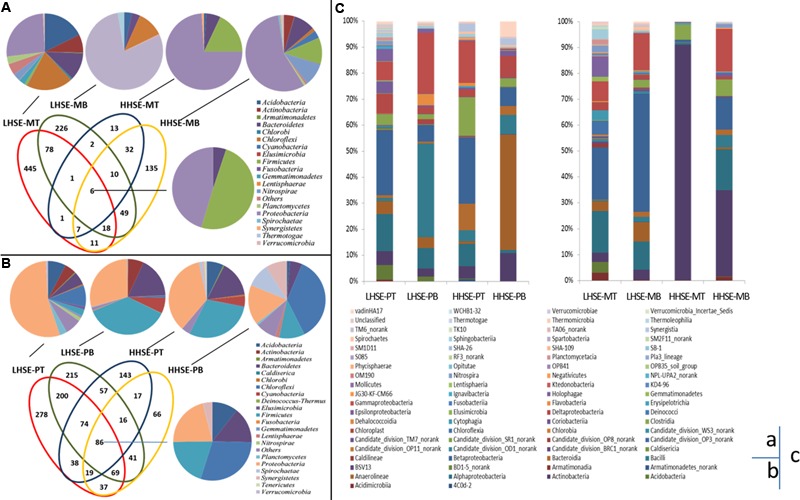
**Microbial community distribution based on 454 pyrosequencing.** Overlapping and individual OTUs displayed using a Venn diagram. Class affiliations of OTUs shared by 2, 3, or 4 manholes and sewerage pipes are shown as a pie chart **(A,B)**. Histogram showing the microbial community distribution of each site at the genus level **(C)**. OTUs are defined by 3% distances.

Performance of 454 pyro-sequencing on the urban sewerage system samples yielded between 5,572 and 16,295 sequences corresponding to the V3 region in each sample, 104,849 sequences were generated from the eight samples, and 83,111 trimmed sequences were obtained, which accounted for 79.27% of the total sequences. **Figures 4A,B** present the unique bacterial OTUs for each sample site and those shared by two, three or four sites in the sewerage system. Core communities are consisting of six OTUs [dominated by *Firmicutes* (49.56%) and *Proteobacteria* (45.32%)] and 86 OTUs (dominated by *Proteobacteria, Firmicutes, Chloroflexi, Bacteroidetes*, and *Acidobacteria*) in the manhole and sewerage pipes, respectively. Unique OTUs were found in individual samples from the LHSE-MT and the LHSE-MB sites. These unique OTUs contributed 66.34 and 46.30% of genetic information, respectively, while providing less than 13.44% contribution from the HHSE manhole (**Figure 4B**). There was a larger proportion of *Chloroflexi* at the LHSE manhole than at the HHSE manhole, while *Acidobacteria* and *Actinobacteria* were mainly detected at the LHSE-MT site. However, the unique OTUs found in each sample from the sewerage pipe contributed less than 26.08% of the total genetic information. Unique OTUs present in the manhole are more possible to represent the distinct microbial community structures in response to the sulfur cycles than those present in the sewerage pipeline.

The samples collected from different positions in the sewerage system exhibited different taxonomic compositions (**Figure 4C**). For example, there were significant variations in *Actinobacteria* population between the manhole and the pipeline at the HHSE site, whereas there was little variation in these bacterial populations at the LHSE site (less than 5.32%). *Actinobacteria* accounted for 90.91% of the composition of the sample from the HHSE-MT site, 33.50% of the composition of the sample from the HHSE-MB site, and only 4.7 and 10.81% of the compositions of the samples collected from the HHSE-PT and HHSE-PB sites, respectively. *Proteobacteria* appeared more frequently at the bottom of the manhole and at the top of the pipe (72.8 and 47.1% of the compositions of the samples taken from the bottom of the manhole and 58.11 and 53.6% of the compositions of the samples taken from the crown of the connected sewerage pipe). Furthermore, the class of Δ-*proteobacteria*, which includes most SRB species, was present at a smaller proportion in the manhole (2.43% at the LHSE site and 0.49% at the HHSE site on average) than that in the sewerage pipes (5.05% at the LHSE site and 5.54% at the HHSE site on average).

### Characterizing of the Functional Microbial Community Responsible for Sulfur Cycle

**FIGURE 5:**
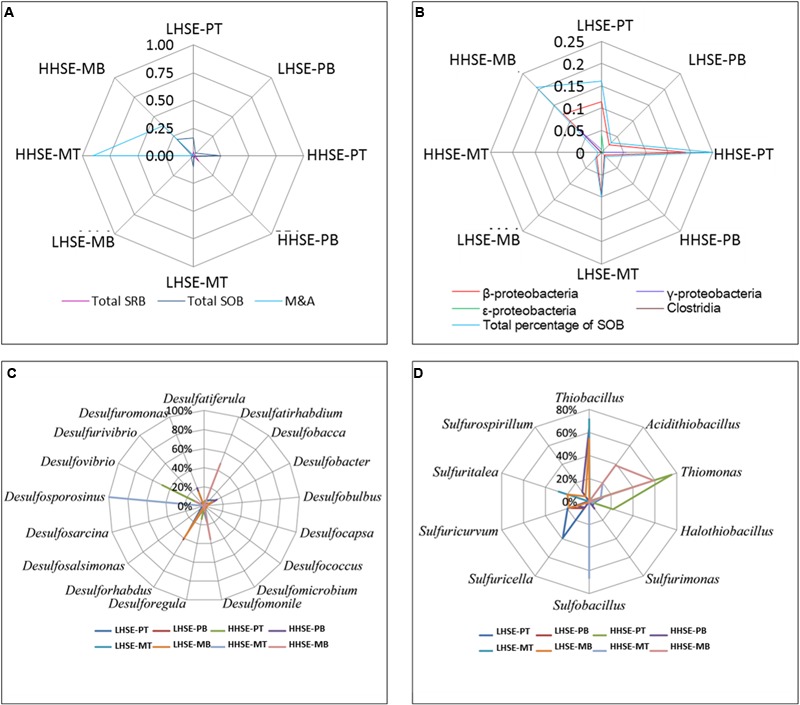
**Microbial quantity-fingerprints of SRB, SOB, and M&A populations at each sampling site (A,B)** and relative proportions of the predominant SRB and SOB species at the genus level for each sample **(C,D)**.

There was a greater proportion of functional bacteria capable of processing sulfur cycle in sewerage pipes compared to manholes, and the total proportion of SOB was higher than that of SRB, whereas there was a greater variety in SRB species (**Figure 5A**). The composition of the SOB community mainly included β-, γ-, and 𝜀-*proteobacteria* and *Clostridia* (**Figure 5B**). There was a higher proportion of SOB at the top of the pipe (HHSE-PT, 25.13%) and the bottom of the manhole (HHSE-MB, 20.63%) (**Figure 5D**), consistent with results previously reported by Satoh et al. (2009). *Thiomonas* and *Halothiobacillus* take up high proportions of SOB population at the HHSE sites. *Thiomonas* survived most frequently at the bottom of manhole and the top of pipeline, which accounted for 59.00% of the SOB population at the in HHSE-MB site and 75.36% of the SOB population at the HHSE-PT site. *Thiobacillus* accounted for the largest proportion of the SOB population at the LHSE-MT (71.57%) and the LHSE-MB (54.55%) sites in the manhole. Correspondingly, a study reported by Mori et al. (1992) showed that sewerage pipes provide suitable conditions for the growth of *Thiobacillus* (SOB) (Nica et al., 2000). Different SOB species might exist at different stages of corrosion, which are accompanied by different pH levels (Okabe et al., 2007). The M&A species, which mainly existed in the environment with relative low pH, were dominant in the HHSE manhole (shown in **Figure 5A**), forming 90.25% of the bacterial population at the top of the manhole and 38.50% of the population at the bottom of the manhole, respectively.

There was a greater proportion of SRB in the sewerage pipes than in the manholes, and the bottom of the sewerage pipes possessed the largest proportions of SRB (shown in **Figure 5A**). A higher proportion of SRB existed at the HHSE-PB site (7.31%) compared to the LHSE site (LHSE-PB 1.74%, LHSE-PT 3.87%). Only one species of *Desulfosporosinus* existed at the top of the HHSE manholes, and no SRB species were detected at the top of the LHSE manhole (**Figure 5C**). However, *Desulforhabdus* accounted for 39.33% of the population at the LHSE site, while *Desulfatirhabdium* accounted for 47.46% of the population at the HHSE site in the bottom of the manhole. *Desulforhabdus* was also detected frequently in the pipeline and accounted for a large proportion of the community at the bottom of the HHSE pipe. Moreover, *Desulfobacter* spp., which was a strictly anaerobic chemoorganotrophic SRB, was also dominant at the bottom of the HHSE pipeline (**Figure 5C**) (Gomez-Alvarez et al., 2012).

### Relationships between Functional Microbial Group and Dynamic Environmental Factors

**FIGURE 6:**
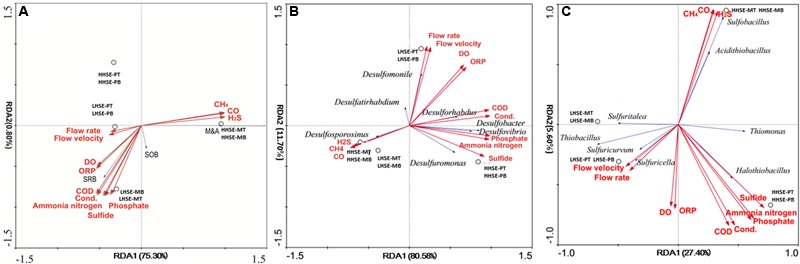
**Redundancy analysis (RDA) of different functional groups, including SRB, SOB, and M&A communities exposed to fluctuating of environmental factors **(A)**.** RDA of the functional species and the dynamic environmental factors **(B/C)**. Dynamic environmental factors were calculated based on standard deviations of 24-h-monitoring data collected at the sampling sites.

Functional microbial groups and pre-selected indicative SRB and SOB species were examined with gas-phase and liquid-phase main dynamic environmental factors by RDA and CCA analysis (base on the length of gradient of DCA-axis 1 of species) (**Figures 6A–C**). M&A species and gas-phase dynamic environmental factors (H_2_S, CO, and CH_4_) appeared to have a strong correlation. Correspondingly, there was a strong correlation between the presence of SRB and different dynamic liquid-phase environmental factors, whereas the SOB were divided into two groups, which might exist in high H_2_S exposure environment (*Acidithiobacillus, Sulfobacillus, Sulfurospirillum, Thiomonas*, and *Halothiobacillus*) and low H_2_S exposure environment (*Sulfuritalea, Thiobacillus, Sulfuricurvum, Sulfurimonas*, and *Sulfuricella*), respectively. Spearman analysis of the SOB species and environmental parameters could also explain the above results partly (Supplementary Table S2). As shown in **Figure 6B** and the spearman analysis (Supplementary Table S1), the presence of *Desulforhabdus* (SRB) and *Desulfobacter* (SRB) appeared to be most strongly positively correlated with different dynamic liquid-phase environmental factors, while the presence of *Desulfosporosinus* (SRB) was strongly positively correlated with various dynamic gas-phase environmental factors. According to the results in **Figure 6C** and the spearman analysis (Supplementary Table S2), the presence of *Thiomonas* (SOB) and *Halothiobacillus* (SOB) was strongly correlated with various dynamic liquid-phase environmental factors, while the presence of *Sulfobacillus* (SOB) and *Acidithiobacillus* (SOB) was strongly correlated with various dynamic gas-phase environmental factors. Moreover, the spatial structures within the manhole and pipeline also had notable correlations with the distributions of the functional microbial communities. *Desulforhabdus* (SRB), *Desulfobacter* (SRB), and *Desulfuromonas* (SRB) were more correlated with the HHSE-PT/B site, while the *Desulfomonile* (SRB), *Desulfurivibrio* (SRB), and *Desulftirhabdium* (SRB) were more correlated with the LHSE-PT/B site. Moreover, *Desulfosporosinus* (SRB) was more likely to reproduce in the manhole. Additionally, the SOB species including *Thiomona, Halothiobacillus, Sulfobacillus*, and *Acidithiobacillus* were correlated with the high H_2_S exposure site, while *Sulfuritalea, Thiobacillus, Sulfuricurvum, Sulfurimonas*, and *Sulfuricella* were more likely to survive in the LHSE environment.

## Discussion

### Influence of Environmental Factors on the Characteristics of Microbial Community

The change of sewerage system structure and fluctuations in source wastewater quality, wastewater quality and quantity cause dynamic changes in environmental factors at different sites within the system. These fluctuations will significantly result in the accumulation and shift of functional microbial community, which participated in the sulfur cycle within the sewerage system (**Figure 6**). The richness of microbial communities and their distributions of functional bacteria reveal the specific rules in response to environmental conditions at different locations within sewerage systems. Microbial diversity was higher in the HHSE sample than in the LHSE sample in this study. The calculated Shannon-Wiener indices showed that a higher diversity in the sewerage pipe sample compared to the manhole. This finding shown different from previous results showing that there is higher microbial diversity in manholes compared to the tops of sewerage pipes (Santo Domingo et al., 2011). The spatial distribution and variation of functional microbial communities also showed significant feature by the taxonomic composition analysis. In previous studies, *Proteobacteria* were predominantly detected within the concrete samples collected from the sewer, accounting for 77 and 75% of the total clones (Okabe et al., 2007; Satoh et al., 2009). Whereas, the *Actinobacteria* (90.91% of the population at the HHSE-MT site and 33.50% of the population at the HHSE-MB site) and *Proteobacteria* (72.8% of the population at the HHSE-MB site and 47.1% of the population at the LHSE-MB site and 58.11% of the population at the HHSE-PT site and 53.6% of the population at the LHSE-PT site) were dominant in this study and exhibited significant differences in localization within the actual sewerage system.

### Correlation of Environmental Factors and Distribution Characteristics of Sulfur-Functional Bacteria

Specific locations within the manholes and pipelines also critically influenced the compositions of functional microbial communities. The distributions of SRB and SOB communities were notably different among the different sampling locations. In the manhole, *Desulfobulbus* sp. and *Desulfobacter postgatei* spp. existed at all samping sites, which might present that both genera had highly tolerant for oxygen concentration (Gutierrez et al., 2008; Colin et al., 2012). It shown different between the distribution of SRB and SOB, and results are consistent with the distribution of phylotypes measured in a previous metagenomic analysis, in which SRB were found be mainly exist at the bottoms of pipelines, while SOB predominated at the tops of sewerage pipes (Gomez-Alvarez et al., 2012). Environmental factors dynamically change throughout the sewerage systems and significantly affect the distributions and localizations of dominant functional microbes within sewerage systems. H_2_S exposure would be considered as a key typical indicator for the potential corrosion in the actual sewerage system (Pagaling et al., 2014). *Thiomonas* and *Halothiobacillus* (dominant SOB) and *Desulforhabdus* and *Desulforbacter* (dominant SRB) survived well at the HHSE-PT/B site, and their presence was positively correlated with the concentration of sulfide. Significant correlation were found between *Desulfosporosinus* and H_2_S concentration, suggesting that the *Desulfosporosinus* was prone to be survived in the manhole and it was sensitive to H_2_S in manhole.

In contrast, shifts in critical microbial community will essentially be able to determine the potential occurrence of the sulfur cycle in different spatial locations within the sewerage system. The presence of *Thiomonas* and *Halothiobacillus* (SOB) and *Desulforhabdus* and *Desulforbacter* (SRB), which has been reported to be implicated in the corrosion of concrete surface (Santo Domingo et al., 2011), was related to various dynamic liquid-phase dynamic environmental factors, while the presence of *Sulfobacillus* and *Acidithiobacillus* (SOB) was related to gas-phase environmental factors. According to the total proportions of SOB and the dominant SOB (*Thiomonas*), it could be suggested that the region of sewerage were the possible places with obvious sulfur cycle reaction. The critical bacteria would further reveal a more distinct reaction related to the sulfur cycle within the sewerage system. MICC has been extensively observed in both pipes and pipeline junctions (Roberts et al., 2002). Due to the acidophilic nature of the M&A group, it could be an indicator of the late period of the H_2_S-H_2_SO_3_ process that occurs during the sulfur cycle (Okabe et al., 2007; Pagaling et al., 2014). If SOB and M&A populations are present in higher proportions in pipe crowns relative to pipe bottoms and there are much higher numbers of SOB species than M&A species, especially in pipe crowns, then MICC is likely at an early stage. In the current study, larger proportion of M&A species were detected and existed in the HHSE manhole, accounting for up to 90.25 and 38.50% of the microbial species found at the top and bottom regions of the manhole, respectively. This finding may implicate a potential serious corrosion because the HHSE manhole might prefer to form an eosinophilic environment by M&A group.

## Conclusion

Species distributions within microbial communities were screened to reveal the microbial diversity, the taxonomic distribution of OTUs, the class-level microbial community structures, and the genus-level microbial community structures that exist under typical environmental fluctuations in a sewerage system. Microbial diversity was found to be higher at HHSE sites compared to LHSE sites and was also higher in sewerage pipes compared to sewer manholes. Unique OTUs could be considered to investigate the distinct microbial community distributions at different locations within the sewerage system. Functional microbial community (SRB, SOB, and M&A) could differentially affect sulfur processing under different environmental conditions. *Thiomonas* (the dominant SOB in this study), *Mycobacterium, Desulforhabdus*, and *Desulfobacter* (the dominant SRB in this study) could therefore be critical species in the sulfur cycle in sewerage systems. The current study provided insights into the roles of different microbial communities in sulfur cycle in sewerage system.

## Author Contributions

QD was in charge of completing the experiment and analyzed the data, and draft the article; YL had proposed the assumption of the paper, revised the draft and do the final version approval; HS did the manuscript revision and gave suggestions on the manuscript structure.

## Conflict of Interest Statement

The authors declare that the research was conducted in the absence of any commercial or financial relationships that could be construed as a potential conflict of interest.

## Footnotes

1 http://qiime.org/

2 http://drive5.com/uparse/

## Supplementary Material

FIGURE S1 **Sulfate-reducing bacteria- denaturing gradient gel electrophoresis (SRB-DGGE) fingerprints of samples collected from different spatial locations at each sampling site (MA, MB, MC, MD, and ME represent manhole samples; PA, PB, PC, PD, and PE represent pipeline samples; 1, 2, 3, 4, and 5 represent different spatial locations)**.

FIGURE S2 **Heatmap showing the relative abundance of each of the OTUs in each sample.** Sample clustering results are shown at the top of the figure. The phylogenetic relationships found to exist between the species are shown on the left side of the figure.
