# Supplementary figures and images for: Microbial Character Related Sulfur Cycle under Dynamic Environmental Factors Based on the Microbial Population Analysis in Sewerage System
Source: Front Microbiol. 2017 Feb 14;8:64. doi: 10.3389/fmicb.2017.00064 (PMC5306501; doi:10.3389/fmicb.2017.00064)

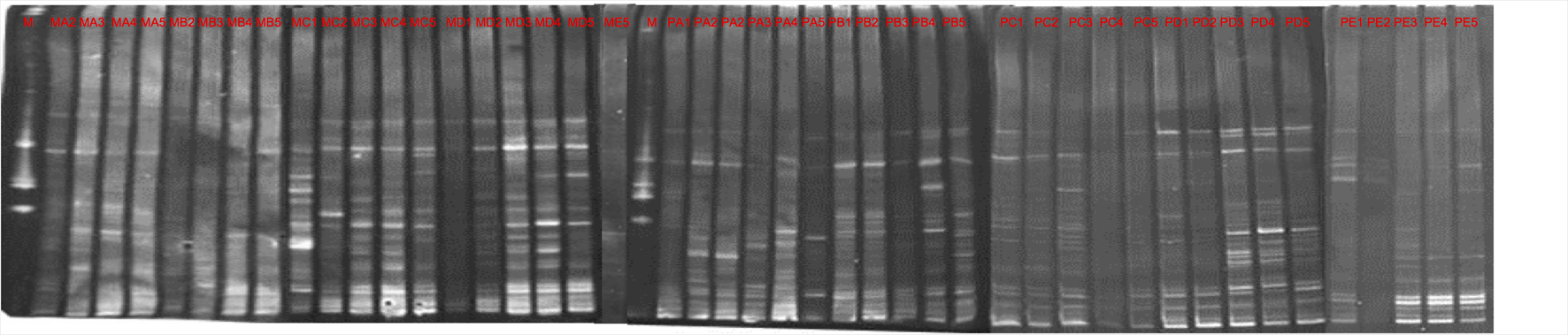

Supplement: FIGURE S1 — Sulfate-reducing bacteria- denaturing gradient gel electrophoresis (SRB-DGGE) fingerprints of samples collected from different spatial locations at each sampling site (MA, MB, MC, MD, and ME represent manhole samples; PA, PB, PC, PD, and PE represent pipeline samples; 1, 2, 3, 4, and 5 represent different spatial locations). [file Image_1.TIFF]

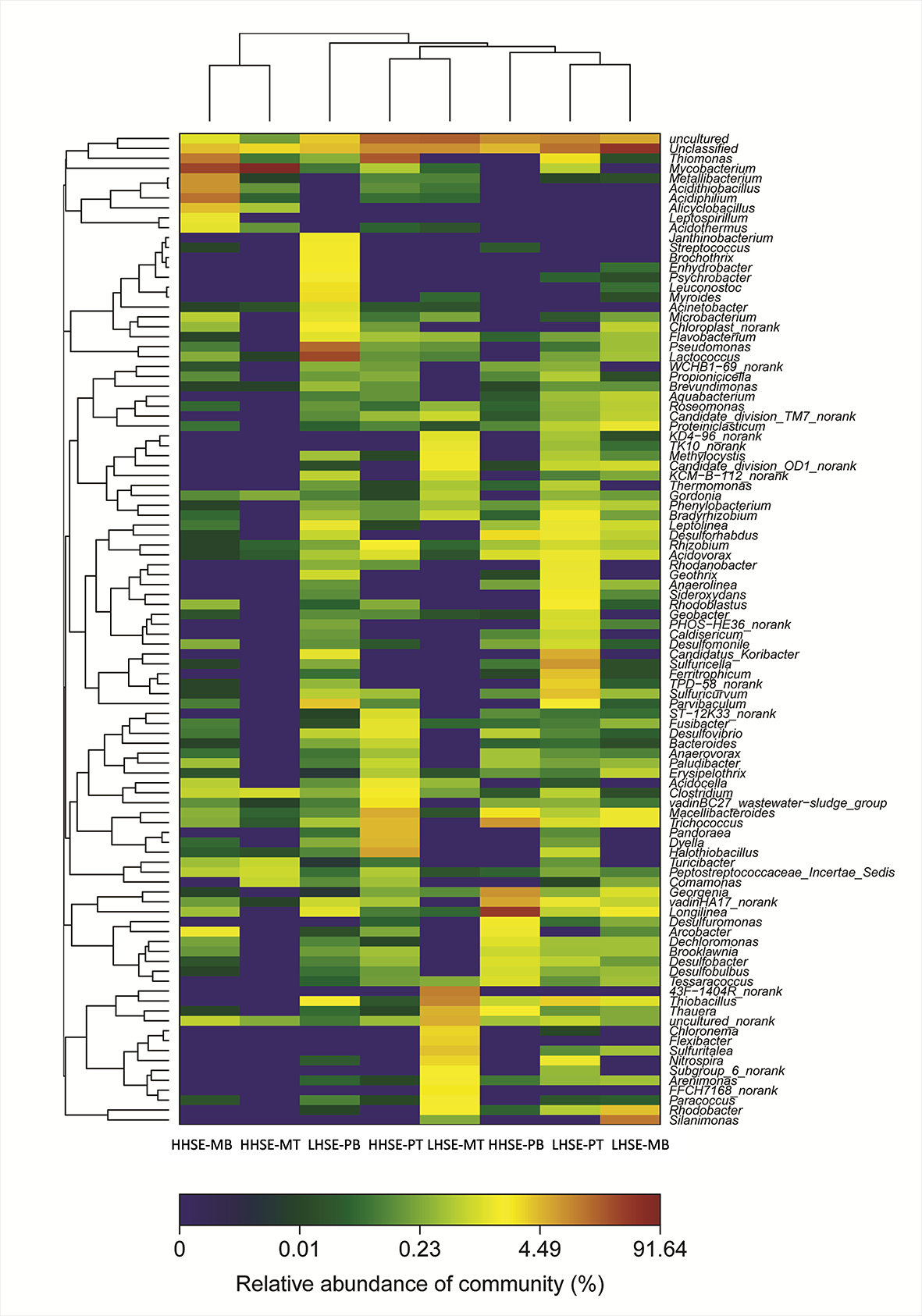

Supplement: FIGURE S2 — Heatmap showing the relative abundance of each of the OTUs in each sample. Sample clustering results are shown at the top of the figure. The phylogenetic relationships found to exist between the species are shown on the left side of the figure. [file Image_2.TIF]
