# Supplementary material for: Microbial Character Related Sulfur Cycle under Dynamic Environmental Factors Based on the Microbial Population Analysis in Sewerage System
Source: Front Microbiol. 2017 Feb 14;8:64. doi: 10.3389/fmicb.2017.00064 (PMC5306501; doi:10.3389/fmicb.2017.00064)
Supplement: Supplementary file 3 [file Table_1.PDF]

Table S1 Spearman correlation of SRB species and the environmental parameters

Spearman correlation	H_2S	CH_4	CO	DO	COD	Sulfide	Ammonia-N
Desulforhabdus	-0.55	-0.55	-0.55	0.61	0.45	0.45	0.45
Desulfobacter	-0.78	-0.78	-0.78	0.65	0.80	0.80	0.80
Desulfuromonas	-0.51	-0.51	-0.51	0.26	0.51	0.51	0.51
Desulfomonile	-0.34	-0.34	-0.34	0.52	0.31	0.31	0.31
Desulfatirhabdium	-0.12	-0.12	-0.12	0.37	0.18	0.18	0.18
Desulfurivibrio	-0.71	-0.71	-0.71	0.82	0.59	0.59	0.59
Desulfosporosinus	0.53	0.53	0.53	-0.36	-0.36	-0.36	-0.36
