# Supplementary material for: Microbial Character Related Sulfur Cycle under Dynamic Environmental Factors Based on the Microbial Population Analysis in Sewerage System
Source: Front Microbiol. 2017 Feb 14;8:64. doi: 10.3389/fmicb.2017.00064 (PMC5306501; doi:10.3389/fmicb.2017.00064)
Supplement: Supplementary file 4 [file Table_2.PDF]

Table S2 Spearman correlation of SOB species and the environmental parameters

Spearman correlation	H_2S	CH_4	CO	DO	COD	Sulfide	Ammonia-N
Acidithiobacillus	0.70	0.70	0.70	-0.59	-0.40	-0.40	-0.40
Thiomonas	-0.09	-0.09	-0.09	0.30	0.22	0.22	0.22
Halothiobacillus	-0.40	-0.40	-0.40	0.61	0.45	0.45	0.45
Sulfurimonas	-0.71	-0.71	-0.71	0.82	0.59	0.59	0.59
Sulfobacillus	0.81	0.81	0.81	-0.54	-0.54	-0.54	-0.54
Sulfuricella	-0.55	-0.55	-0.55	0.71	0.34	0.34	0.34
Sulfuricurvum	-0.70	-0.70	-0.70	0.78	0.47	0.47	0.47
Sulfuritalea	0.09	0.09	0.09	-0.27	-0.44	-0.44	-0.44
Sulfurospirillum	-0.25	-0.25	-0.25	-0.05	0.49	0.49	0.49
